# Periprocedural Outcomes of VT Ablation in Ischemic Compared to Non‐Ischemic Dilated Cardiomyopathy

**DOI:** 10.1111/anec.70126

**Published:** 2025-11-04

**Authors:** Sanchit Duhan, Shafaqat Ali, Thannon Alsaeed, Manoj Kumar, Bijeta Keisham, Sanjay S. Mehta, Naveed A. Adoni, Mbu Mongwa, Benjamin J. Rhee, Anuj Garg

**Affiliations:** ^1^ Department of Cardiology, Carle Foundation Hospital University of Illinois Urbana‐Champaign Champaign Illinois USA; ^2^ Department of Internal Medicine Louisiana State University Shreveport Louisiana USA; ^3^ Department of Internal Medicine Cook County Hospital Chicago Illinois USA; ^4^ Department of Internal Medicine Shandong Second Medical University Weifang Shandong China

**Keywords:** catheter ablation, ischemic cardiomyopathy, non‐ischemic dilated cardiomyopathy, ventricular tachycardia

## Abstract

**Background:**

Patients with structural heart disease undergoing catheter ablation (CA) for VT have shown higher procedural‐related adverse events. However, periprocedural outcomes comparing CA for VT in different cardiomyopathies are not well known. We aim to study short‐term outcomes of CA in ischemic (ICM) compared to non‐ischemic dilated cardiomyopathy (NIDCM).

**Methods:**

The national readmission database (2016–2020) was used to identify hospitalizations for CA for VT. Cohorts were stratified based on underlying cardiomyopathy. A Propensity Score Matching (PSM) model matched ICM to NIDCM patients. Pearson's Chi‐squared test was applied to PSM‐matched cohorts to compare outcomes.

**Results:**

Among 7081 hospitalizations for VT ablation, 17.5% of patients had underlying NIDCM, while 82.5% of patients had ICM. On a PSM analysis (*N*: 3534), ICM patients had higher incidences of sudden cardiac arrest (SCA) (7.9% vs. 5.6%, *p* < 0.001), major adverse cardiac events (11.1% vs. 9%, *p*: 0.006), and cardiogenic shock (10.8% vs. 8.5%, *p*: 0.001). Interestingly, NIDCM patients were found to have much higher rates of pericardial complications (6.09% vs. 1.90%, *p* < 0.001), while the mortality difference was not significant (*p* > 0.05). From 2016 to 2020, in‐hospital mortality rates have not changed significantly in ICM and NIDCM cases admitted for VT ablation (*p*‐trend > 0.05); however, there was a decreasing trend of SCA cases in NIDCM hospitalizations (8.7%–3.4%, *p*‐trend: 0.028). NIDCM patients had higher readmission rates at 30 days (18% vs. 15.5%, *p*: 0.01), 90‐day (32.3% vs. 29.6%, *p*: 0.041), and 180‐day (44% vs. 38.2%, *p*: 0.001).

**Conclusion:**

VT ablation in ICM patients was associated with higher non‐fatal periprocedural events. NIDCM patients showed higher all‐cause readmission rates.

## Graphical abstract

VT ablation in ICM is associated with higher periprocedural adverse events except pericardial complications, which are higher in NIDCM. Created using Biorender.com. C‐shock, cardiogenic shock; ICM, ischemic cardiomyopathy; MACE, major adverse cardiovascular events; NIDCM, non‐ischemic dilated cardiomyopathy; PSM‐propensity score matching; SCA‐sudden cardiac arrest; VT, ventricular tachycardia.

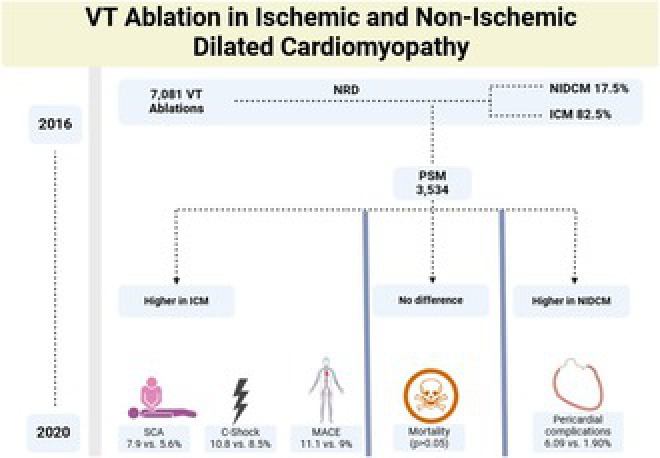

## 1 Introduction

Catheter ablation (CA) is an effective method to reduce ventricular tachycardia (VT) recurrence and implantable cardioverter defibrillator therapies (anti‐tachycardia pacing and shocks) (Stevenson et al. 2008; Kuck et al. 2010). CA is typically guided by pace mapping and identifying abnormal electrograms within the arrhythmogenic substrate (Reddy et al. 2007; Marchlinski et al. 2000). The critical circuit components (e.g., VT isthmus) and ablation approaches differ between ischemic cardiomyopathy (ICM) and non‐ischemic dilated cardiomyopathy (NIDCM) (Shirai et al. 2019). Accordingly, one would expect different outcomes in these patient groups. Most prior studies discuss the VT recurrence rates in ICM versus NIDCM and direct comparisons of post‐ablation complications are scarce (Kanagaratnam et al. 2022; Sciria et al. 2022). Consequently, we performed this nationwide readmission database (NRD) analysis to evaluate VT ablation outcomes in ICM and NIDCM patients.

## 2 Methods

### 2.1 Study Design and Population

This study utilized data from the NRD spanning 2016–2020. The NRD, managed by the Agency for Healthcare Research and Quality (AHRQ) (Anon 2025a), provides insights into approximately 35 million weighted hospitalizations across the United States. It represents a nationally representative administrative database covering 62.2% of all hospital discharges and readmissions.

Patients admitted with ventricular tachycardia (VT) were identified using the International Classification of Diseases, Tenth Edition, Clinical Modification (ICD‐10‐CM) codes. ICD‐10 Procedural Coding System (ICD‐10‐PCS) codes were employed to identify patients who underwent catheter ablation (CA) and classified patients into ICM and NIDCM groups (Anon 2025b). The specific ICD‐10 codes used for patient selection and outcome measures are detailed in Table S1.

Trend analysis was performed on the entire study cohort for in‐hospital mortality, resource utilization, and interventions during the initial hospitalization. Patients were categorized based on cardiomyopathy type into ICM and NIDCM cohorts for outcome analysis.

Patients under the age of 18 and duplicate records were excluded. The final cohort was divided into two groups: VT CA patients with ICM and VT CA patients with NIDCM. A unique identifier code was assigned to each individual case. Variables such as length of stay (LOS) and time to intervention were used to calculate readmission timelines. Data from index admission were used for analysis. As the NRD is annualized, only patients admitted within the same calendar year could be tracked. To ensure complete follow‐up, data from the first 11, 9, and 6 months of each year were sequentially included for 30‐, 90‐, and 180‐day readmissions, respectively. Observations with cell counts below 11 were omitted, following HCUP reporting guidelines.

### 2.2 Baseline Characteristics

We selected adult patients (age ≥ 18 years) hospitalized with VT between 2016 and 2020. Baseline characteristics, including age, sex, and co‐morbidities, were analyzed. Hospital‐specific variables such as bed size, teaching status, and urban–rural classification were also considered.

### 2.3 Study Outcomes

The primary outcome was the comparison of in‐hospital mortality between VT patients with ICM and NIDCM. Secondary outcomes included additional complications during hospitalization, such as sudden cardiac arrest (SCA), acute stroke, major adverse cardiovascular events (MACE), defibrillation, implantable cardioverter defibrillator (ICD) placement, cardiogenic shock, acute congestive heart failure (CHF), pericardial complications, postprocedural bleeding, and respiratory complications. Other measures included length of stay (LOS), adjusted total charges, propensity‐matched 30‐, 90‐, and 180‐day readmission rates, as well as trends in VT CA‐related mortality, sudden cardiac arrest, and ICM among ICM and NIDCM patients (Figure S3). The definitions of all study outcomes are available in Table S2.

### 2.4 Statistical Analysis

Descriptive statistics were applied to summarize continuous and categorical variables. Categorical variables were expressed as percentages and frequencies and compared using Pearson's Chi‐squared test. Based on normality, independent sample *t*‐tests were used for normally distributed data, while Mann–Whitney *U* tests (Wilcoxon rank sum tests) were applied for non‐parametric distributions.

Patient demographics, co‐morbidities, and study outcomes were compared between the ICM and NIDCM cohorts. Little's MCAR test was conducted to assess missing data patterns. A *p*‐value > 0.05 indicated data missing completely at random (MCAR), while a *p*‐value < 0.05 suggested non‐random missing data (MNAR). Missing values were recorded for variables such as Primary Expected Payer, Admission Status, and Median Household Income, with overall missing data remaining below 2%. These were marked as missing and excluded from the analysis.

For outcome analysis, both unadjusted and adjusted odds ratios (ORs) were calculated using univariate and multivariate logistic regression. Adjusted ORs were determined for in‐hospital outcomes, considering significance at *p* < 0.05 and confidence intervals not crossing 1. A univariate screening process (*p*‐value < 0.2 threshold) was used to select variables for the final multivariate regression model. Variance Inflation Factor (VIF) and tolerance (1/VIF) were used to assess multicollinearity, with VIF > 5 and tolerance < 0.2 indicating strong correlations among variables. The covariates included in the regression model are listed in Table S4.

A Propensity Score Matching (PSM) model was employed, using Mahalanobis Distance Matching within a 0.2 caliper to match VT patients with ICM and NIDCM. Variables from the multivariate regression model were included in PSM to verify consistency with logistic regression findings. Pearson's Chi‐squared test was used to compare outcomes in the matched cohorts. The matching criteria, including demographics, disease severity, mortality risk, and 15 baseline co‐morbidities, are detailed in Table S3.

A similar PSM model was performed for 30‐, 90‐, and 180‐day readmission analyses. To mitigate mortality readmission bias, only patients discharged alive were included in the readmission analysis. Using combined data from all years, a multivariable logistic regression model was utilized to generate predictive margins for adjusted trends over time, with year of admission as an independent variable.

Unadjusted trend analysis was conducted using the Cochran‐Armitage test for binary outcomes and either the Jonckheere‐Terpstra test or Cuzick's test for ordered categorical or continuous variables, given the non‐parametric distribution of the study cohort. Total costs were inflation‐adjusted and merged with cost‐charge ratio (CCR) NRD files.

All statistical analyses adhered to Healthcare Cost and Utilization Project (HCUP) regulations regarding stratification, clustering, and weighting. Analyses were conducted using Stata v.18 software (Stata Corp, College Station, TX). Central illustration was created using Biorender.com.

## 3 Results

### 3.1 Demographic and Baseline Characteristics

A retrospective analysis was conducted on a cohort of 7080 hospitalizations for ventricular tachycardia (VT) ablation. A majority (82.5%) of the patients had underlying ischemic cardiomyopathy (ICM), while only 17.5% (*N*: 1238) of the patients had underlying non‐ischemic dilated cardiomyopathy (NIDCM) (Figure S2). ICM patients were found to be older, with a median age of 68 years (interquartile range, IQR: 13 years) compared to a median age of 61 years (IQR: 19 years) of NIDCM patients (*p* < 0.001). Most of these patients (three‐fourths) were admitted electively in both groups with no significant difference (*p*: 0.254). Both groups were similar in demographic factors, including the characteristics of the hospital (urban–rural designation, bed size, and teaching status) and median household income (*p* > 0.05). Patient factors, including the severity of illness and risk of mortality as assessed on All‐Patient Refined Diagnosis Related Groups (APRDRG), were also very similar between the two groups (*p* > 0.05).

**FIGURE 1:**
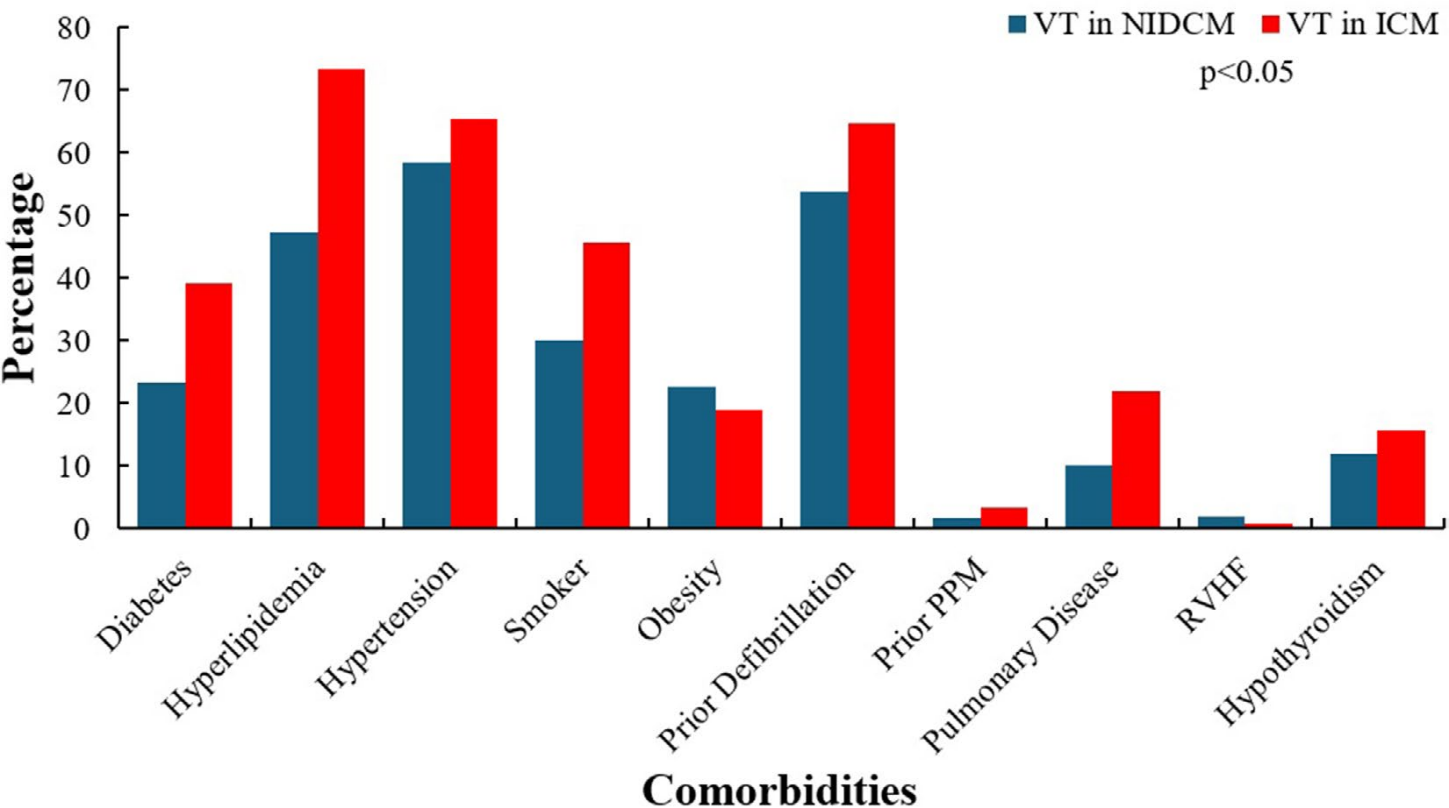
Baseline co‐morbidities. ICM, ischemic cardiomyopathy; NIDCM, nonischemic dilated cardiomyopathy; PPM, permanent pacemaker; RVHF, right ventricular heart failure.

The prevalence of various co‐morbidities differed between the two groups, with ICM patients having a higher prevalence of diabetes (39.1% vs. 23.2%, *p* < 0.001), hyperlipidemia (73.4% vs. 47.3%, *p* < 0.001), hypertension (65.4% vs. 58.5%, *p*: 0.002), smoking history (45.7% vs. 30.1%, *p* < 0.001), CKD stage > 3 (32.5% vs. 27.9%, *p*: 0.039), pulmonary disease (22% vs. 10%, *p* < 0.001), and presence of a defibrillator (64.7% vs. 53.9%, *p* < 0.001) or a permanent pacemaker (3.4% vs. 1.6%, *p*: 0.01). Other co‐morbidities are summarized in Table 1 and Figure 1.

**TABLE 1:** Baseline characteristics and co‐morbidities comparison of VT ablation in ICM and NIDCM.

Baseline characteristics	NIDCM	ICM	*p*
	*N* = 1239	*N* = 5842	
	*N* (%)	*N* (%)	
Age (Median + IQR)			
	61 (19)	68 (13)	< 0.001
Indicator of sex			
Male	984 (79.4)	5237 (89.6)	< 0.001
Female	255 (20.6)	605 (10.4)	
Insurance type			
Medicare	623 (50.3)	4042 (69.3)	< 0.001
Medicaid	156 (12.6)	348 (6)	
Private insurance	416 (33.6)	1157 (19.8)	
Self‐pay	12 (1)	56 (1)	
No charge	8 (0.6)	9 (0.2)	
Other	24 (1.9)	221 (3.8)	
Type of admission			
Non‐elective	912 (73.8)	4453 (76.4)	0.254
Elective	323 (26.2)	1378 (23.6)	
Hospital characteristics: hospital bed size			
Small	43 (3.4)	273 (4.7)	0.619
Medium	206 (16.7)	1043 (17.8)	
Large	989 (79.9)	4526 (77.5)	
Hospital characteristics: control/ownership of hospital			
Government, nonfederal	156 (12.6)	660 (11.3)	0.354
Private, not‐profit	985 (79.6)	4614 (79)	
Private, invest‐own	96 (7.8)	569 (9.7)	
Hospital characteristics: teaching status of urban hospitals			
Metropolitan non‐teaching	94 (7.6)	413 (7.1)	0.71
Metropolitan teaching	1125 (90.9)	5359 (91.7)	
Non‐metropolitan hospital	18 (1.5)	70 (1.2)	
Hospital characteristics: hospital urban–rural designation			
Large metropolitan areas with at least 1 million residents	831 (67.2)	3774 (64.6)	0.063
Small metropolitan areas with less than 1 million residents	388 (31.4)	1998 (34.2)	
Micropolitan areas	16 (1.3)	70 (1.2)	
Non‐metropolitan hospital	2 (0.2)	0 (0)	
Admission day is a weekend			
Mon—Fri	1000 (80.8)	4779 (81.8)	0.578
Sat—Sun	238 (19.2)	1063 (18.2)	
Transfer flag indicating combination of discharges involve same day events			
Not a transfer or other same‐day stay	1074 (86.7)	5019 (85.9)	0.59
Transfer involving two discharges from different hospitals	94 (7.6)	543 (9.3)	
Same‐day stay involving two discharges from different hospitals	43 (3.5)	172 (3)	
Same‐day stay involving two discharges at the same hospitals	16 (1.3)	56 (1)	
Same‐day stay involving three or more discharges at the same or different hospitals	11 (0.9)	52 (0.9)	
Median household income national quartile for patient ZIP Code			
0–25th percentile	331 (27.3)	1428 (24.8)	0.078
26th–50th percentile	278 (23)	1661 (28.8)	
51st–75th percentile	353 (29.1)	1514 (26.3)	
76th–100th percentile	249 (20.6)	1163 (20.2)	
Rehab transfer			
Not a combined record or a combined record not involving rehabilitation, evaluation, or other aftercare	1231 (99.4)	5803 (99.3)	0.81
Combined record involving transfer to rehabilitation, evaluation, or other aftercare	7 (0.6)	40 (0.7)	
Patient state is the same as hospital state			
Nonresident	146 (11.8)	797 (13.6)	0.292
Resident	1092 (88.2)	5046 (86.4)	
All patient refined DRG: risk of mortality subclass			
Minor likelihood of dying	21 (1.7)	87 (1.5)	0.072
Moderate likelihood of dying	106 (8.5)	307 (5.3)	
Major likelihood of dying	871 (70.4)	4286 (73.4)	
Extreme likelihood of dying	241 (19.4)	1161 (19.9)	
All patient refined DRG: severity of illness subclass			
Minor loss of function (includes cases with no co‐morbidity or complications)	63 (5.1)	378 (6.5)	0.388
Moderate loss of function	559 (45.2)	2741 (46.9)	
Major loss of function	414 (33.4)	1728 (29.6)	
Extreme loss of function	202 (16.3)	994 (17)	
CHADS‐VASC score			
Low risk	68	0	< 0.001
Moderate risk	150	228	
High risk	1020	5614	
Co‐morbidities			
Diabetes	288 (23.2)	2285 (39.1)	< 0.001
Hyperlipidemia	585 (47.3)	4290 (73.4)	< 0.001
Hypertension	725 (58.5)	3823 (65.4)	0.002
Smoker	373 (30.1)	2670 (45.7)	< 0.001
Obesity	280 (22.6)	1101 (18.8)	0.049
CKD stage over 3	336 (27.1)	1896 (32.5)	0.039
ESRD	24 (1.9)	112 (1.9)	0.985
Prior CVA	3 (0.3)	4 (0.1)	0.227
Prior defibrillation	667 (53.9)	3780 (64.7)	< 0.001
Prior PPM	20 (1.6)	200 (3.4)	0.02
OSA	217 (17.6)	1039 (17.8)	0.902
Pulmonary disease	124 (10)	1284 (22)	< 0.001
Pulmonary hypertension	60 (4.8)	219 (3.8)	0.249
RVHF	23 (1.8)	45 (0.8)	0.018
Hypothyroidism	148 (11.9)	909 (15.6)	0.02
Anemia	43 (3.4)	188 (3.2)	0.797
Liver disease	10 (0.8)	77 (1.3)	0.312
Malnutrition	41 (3.3)	190 (3.2)	0.919

Abbreviations: CKD, chronic kidney disease; CVA, cerebral vascular accident; ESRD, end stage renal disease; ICM, ischemic cardiomyopathy; NIDCM, non‐ischemic dilated cardiomyopathy; OSA, obstructive sleep apnea; PPM, permanent pacemaker placement; RVHF, right ventricular heart failure; VT, ventricular tachycardia.

### 3.2 Outcomes of Unmatched and Propensity‐Matched Cohorts of VT Ablation in Patients With Ischemic Versus Non‐Ischemic Dilated Cardiomyopathy

On crude analysis, VT ablation in ICM and NIDCM had a similar risk profile with no difference in in‐hospital mortality, sudden cardiac arrest (SCA), stroke, cardiogenic shock, respiratory complications, or need for an implantable cardiac defibrillator (ICD) device placement (*p* > 0.05) as shown in Table 2; however, pericardial complications were found to be much higher after VT ablation in patients with NIDCM (6.4% vs. 1.9%, *p* < 0.001).

**TABLE 2:** Crude and propensity matched in‐hospital outcomes of VT ablation in ICM and NCIM.

Outcomes	Crude outcomes			Propensity match outcomes		
	VT in NIDCM	VT in ICM	*p*	VT in NIDCM	VT in ICM	*p*
	*N* (%)	*N* (%)		*N* (%)	*N* (%)	
	*N* = 1239	*N* = 5842		*N* = 3534	*N* = 3534	
Died during hospitalization	53 (4.3)	238 (4.1)	0.806	105 (3.3)	124 (3.9)	0.201
SCA	86 (7)	460 (7.9)	0.478	175 (5.6)	249 (7.9)	< 0.001
Thromboembolic stroke	10 (0.8)	46 (0.8)	0.964	16 (0.5)	26 (0.8)	0.122
MACE	132 (10.6)	655 (11.2)	0.695	283 (9)	349 (11.1)	0.006
ICD placement	193 (15.6)	822 (14.1)	0.36	449 (14.2)	464 (14.7)	0.591
Cardiogenic shock	141 (11.4)	647 (11.1)	0.853	267 (8.5)	342 (10.8)	0.001
Acute CHF	469 (37.9)	2147 (36.8)	0.639	1264 (40.1)	1188 (37.6)	0.05
Pericardial complications	80 (6.4)	114 (1.9)	< 0.001	192 (6.1)	60 (1.9)	< 0.001
Respiratory complications	180 (14.6)	968 (16.6)	0.27	467 (14.8)	509 (16.1)	0.144
Postprocedural bleeding	16 (1.3)	124 (2.1)	0.198	27 (0.9)	73 (2.3)	< 0.001

Abbreviations: CHF, congestive heart failure; ICD, implantable cardioverter‐defibrillator; ICM, ischemic cardiomyopathy; MACE, major adverse cardiac event; NIDCM, non‐ischemic dilated cardiomyopathy; SCA, sudden cardiac arrest; VT, ventricular tachycardia.

On a propensity‐matched analysis (*N* = 6310 with 3155 VT ablations in each group), VT ablation in ICM was found to be associated with a higher risk profile, including an increased incidence of SCA (7.9% vs. 5.6%, *p* < 0.001), cardiogenic shock (10.8% vs. 11.5%, *p* < 0.001) and postprocedural bleeding (2.3% vs. 0.9%, *p* < 0.001) in comparison to patients with NICDM. Notably, pericardial complications were higher in patients with NICDM (6.1% vs. 1.9%, *p* < 0.001) (Central illustration).

**FIGURE 2:**
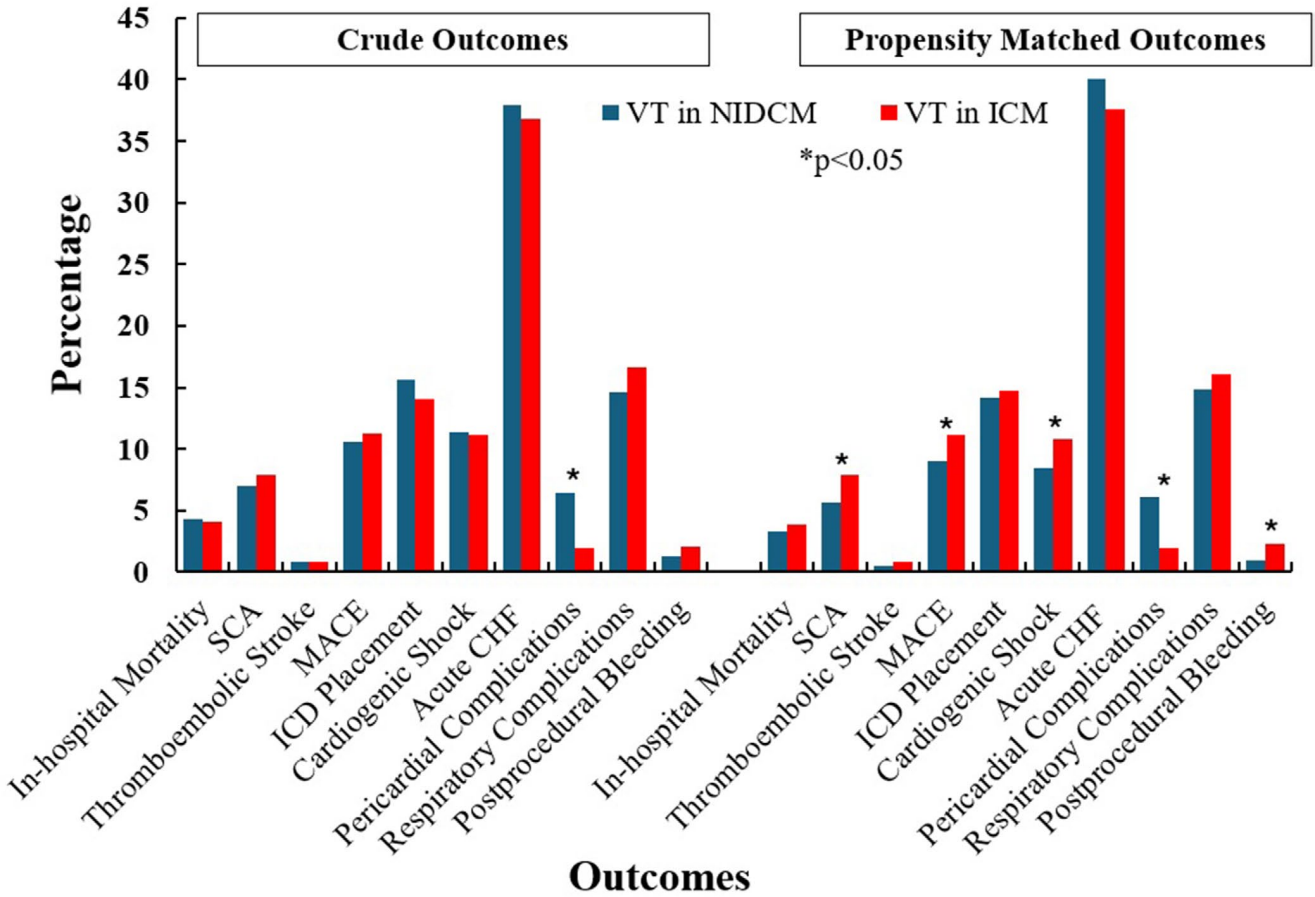
Crude and propensity‐matched outcomes. CHF, congestive heart failure; ICD, implantable cardioverter defibrillator; ICM, ischemic cardiomyopathy; MACE, major adverse cardiovascular events; NIDCM, nonischemic dilated cardiomyopathy; SCA, sudden cardiac arrest; VT, ventricular tachycardia.

Crude and propensity‐matched outcomes are shown in Table 2 and Figure 2.

### 3.3 Multivariate Regression Analysis of Outcomes After VT‐Ablation: Non‐Ischemic Dilated Cardiomyopathy Compared to Ischemic Cardiomyopathy

On a multivariate regression analysis adjusting for potential confounders, VT ablation in ICM was found to be associated with lower incidences of pericardial complications (adjusted odds ratio, aOR: 0.33, 95% CI: 0.20–0.53, *p* < 0.001).

Interestingly, the odds of in‐hospital mortality, sudden cardiac arrest, stroke, cardiogenic shock, respiratory complications, or need for an implantable cardiac defibrillator (ICD) device placement (*p* > 0.05) were similar between the two groups.

**FIGURE 3:**
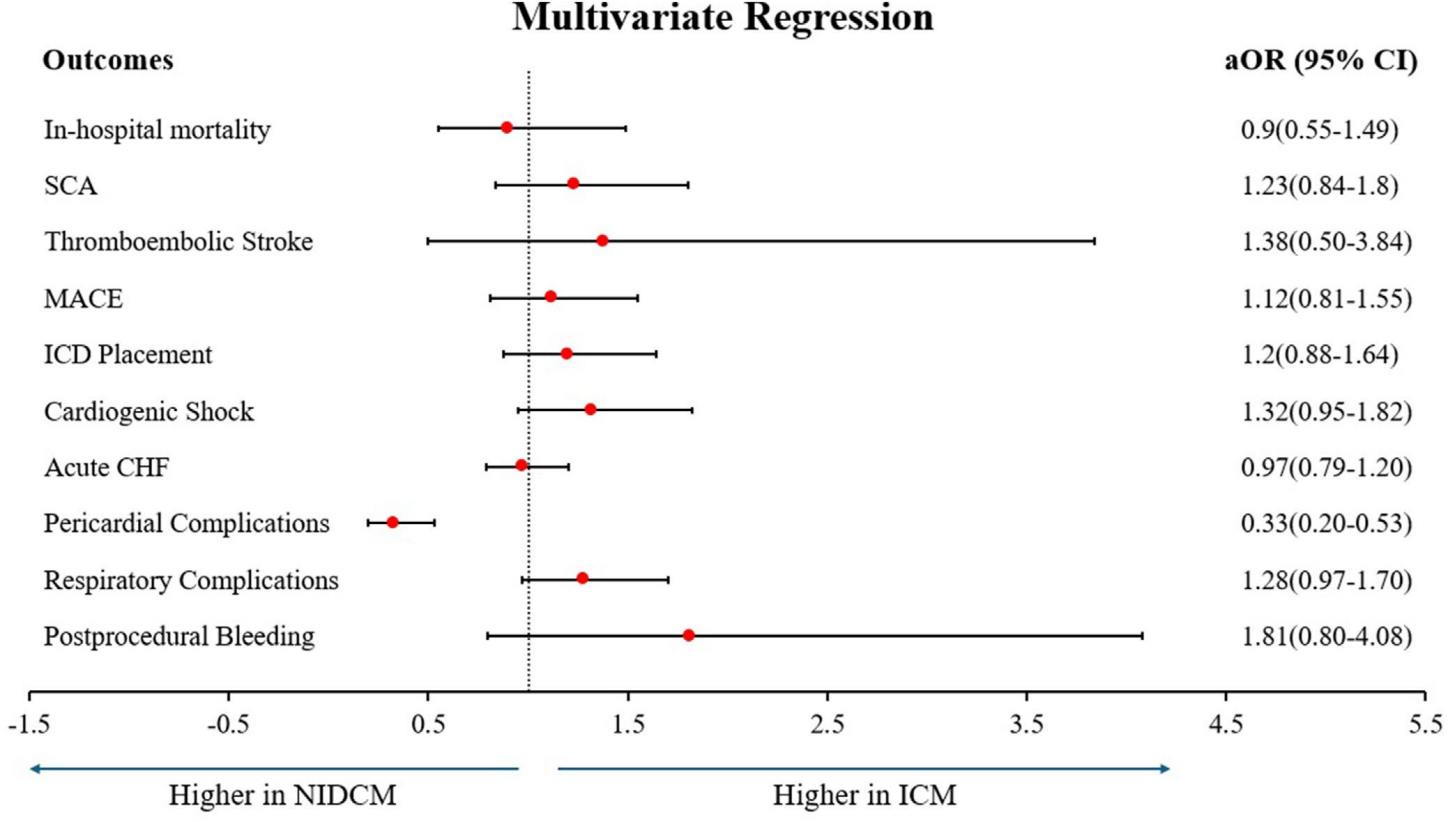
Multivariate regression analysis of the outcomes. CHF, congestive heart failure; ICD, implantable cardioverter defibrillator; ICM, ischemic cardiomyopathy; MACE, major adverse cardiovascular events; NIDCM, nonischemic dilated cardiomyopathy; SCA, sudden cardiac arrest.

Multivariate regression analysis is shown in Table 3 and Figure 3.

**TABLE 3:** Multivariate regression analysis comparing VT ablation in ICM and NIDCM.

In‐hospital outcomes	VT in ICM		
	aOR	95% CI	*p*
Died during hospitalization	0.9	0.55–1.49	0.686
SCA	1.23	0.84–1.8	0.295
Thromboembolic stroke	1.38	0.50–3.84	0.537
MACE	1.12	0.81–1.55	0.489
ICD placement	1.2	0.88–1.64	0.249
Cardiogenic shock	1.32	0.95–1.82	0.096
Acute CHF	0.97	0.79–1.20	0.778
Pericardial complications	0.33	0.20–0.53	< 0.001
Respiratory complications	1.28	0.97–1.70	0.084
Postprocedural bleeding	1.81	0.80–4.08	0.153

Abbreviations: CHF, congestive heart failure; ICD, implantable cardioverter‐defibrillator; ICM, ischemic cardiomyopathy; MACE, major adverse cardiac event; NIDCM, non‐ischemic dilated cardiomyopathy; SCA, sudden cardiac arrest; VT, ventricular tachycardia.

### 3.4 Resource Utilization of Hospitalizations for VT‐Ablations in Patients With ICM and NIDCM

NIDCM patients undergoing VT ablation exhibited an extended length of stay (LOS), with a median length of stay of 5 days (interquartile range; IQR: 6 days) compared to 5 days (IQR: 5 days) for their ICM counterparts (*p* < 0.001). Interestingly, no difference was observed in the median cost of hospitalization between the two groups (*p* > 0.05) (Figure S1).

**FIGURE 4:**
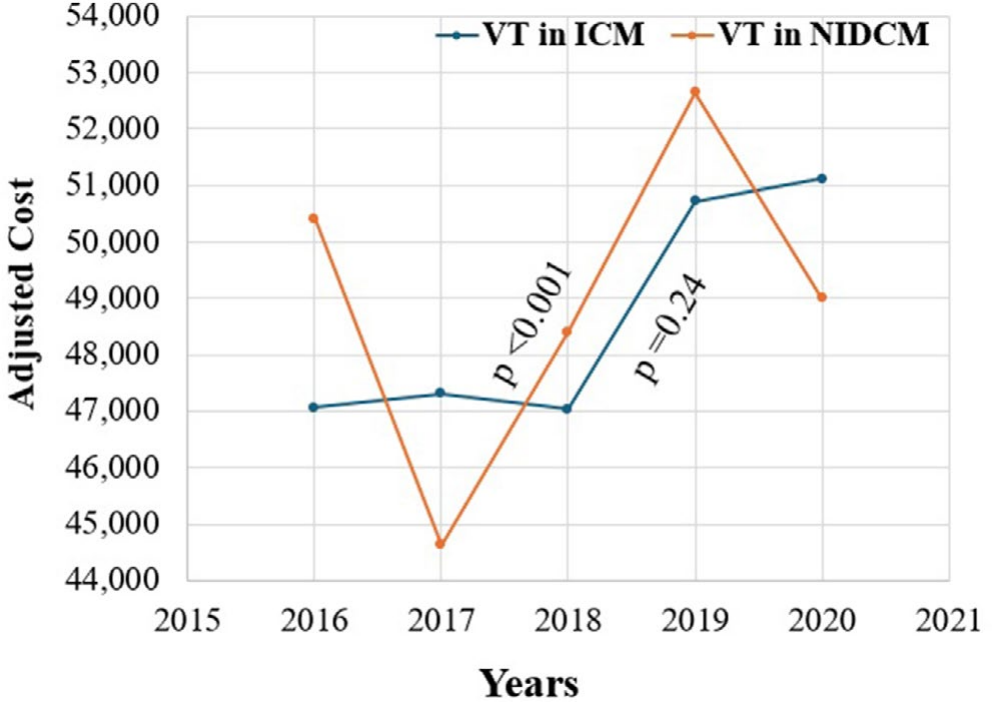
Cost trends over the study period (2016–2020). ICM, ischemic cardiomyopathy; NIDCM, nonischemic dilated cardiomyopathy; VT, ventricular tachycardia.

Resource utilization is shown in Table 5 and Figure 4.

### 3.5 Readmission Rates on Propensity Matched Cohort After VT Ablation in Patients With ICM and NIDCM

On a propensity‐matched cohort, patients with NIDCM were found to have higher 30‐day (18% vs. 15.5%, *p*: 0.01), 90‐day (32.3% vs. 29.6%, *p*: 0.041) and 180‐day (44% vs. 38.2%, *p* < 0.001) all‐cause readmission rates in comparison to ICM counterparts as shown in Table 4. Readmission rates due to heart failure in these cohorts are presented in Table S5.

**TABLE 4:** Readmission rates on propensity matched cohort.

Readmission rates on propensity matched cohort			
30 day readmissions	VT in ICM	VT in NIDCM	*p*
	*N* = 2923	*N* = 2923	
	*N* (%)	*N* (%)	
Readmits	453 (15.5)	527 (18)	0.01
90 day readmissions	VT in ICM	VT in NIDCM	*p*
	*N* = 2367	*N* = 2367	
	*N* (%)	*N* (%)	
Readmits	700 (29.6)	765 (32.3)	0.041
180 day readmissions	VT in ICM	VT in NIDCM	*p*
	*N* = 1591	*N* = 1591	
	*N* (%)	*N* (%)	
Readmits	608 (38.2)	700 (44)	0.001

Abbreviations: ICM, ischemic cardiomyopathy; NIDCM, non‐ischemic dilated cardiomyopathy; VT, ventricular tachycardia.

**TABLE 5:** Resource utilization during the index hospitalization and yearly trend (2016–2020).

Resource utilization	Total adjusted cost		Length of stay	
	VT in ICM	VT in NIDCM	VT in ICM	VT in NIDCM
	Median (IQR)	Median (IQR)	Median (IQR)	Median (IQR)
Index‐hospitalization	48,403 (36,227)	48,129 (42,437)	5 (5)	5 (6)
*p*	0.972		< 0.001	
Yearly trend				
2016	47,059 (33,795)	50,409 (35,404)	5 (5)	5 (7)
2017	47,307 (31,861)	44,633 (32,584)	5 (6)	5 (6)
2018	47,033 (35,992)	48,407 (55,609)	5 (6)	5 (6)
2019	50,718 (42,615)	52,639 (44,909)	5 (5)	6 (8)
2020	51,121 (35,962)	49,006 (37,689)	5 (6)	5 (6)
*p*‐trend	< 0.001	0.245	0.492	0.576

Abbreviations: ICM: ischemic cardiomyopathy; IQR, interquartile range (P75–P25); NIDCM, non‐ischemic dilated cardiomyopathy; VT, ventricular tachycardia.

**TABLE 6:** Yearly trend of mortality, SCA, ICD implantation in VT ablation in ICM and NCIM.

Year	VT in ICM			VT in NIDCM		
	Mortality (%)	SCA (%)	ICD (%)	Mortality (%)	SCA (%)	ICD (%)
2016	4.7	7.3	11.3	3.7	8.7	11.5
2017	3.9	5.9	12.4	3.7	7.5	11.6
2018	4.4	7	12.9	5.5	4.3	11.4
2019	5.3	6.3	16	6.3	4.4	13.2
2020	3.5	4.8	14.8	2	3.4	15.2
*p*‐trend	< 0.001	< 0.001	0.041	< 0.001	< 0.001	0.239

Abbreviations: ICD, implantable cardioverter‐defibrillator; ICM, ischemic cardiomyopathy; NIDCM, non‐ischemic dilated cardiomyopathy; SCA, sudden cardiac arrest; VT, ventricular tachycardia.

### 3.6 Yearly Trend of Mortality, Sudden Cardiac Arrest, ICD Implantation, and Resource Utilization Associated With VT Ablation in Patients With ICM and NIDCM

The median length of stay after VT ablation has remained unchanged regardless of the underlying cardiomyopathy over the years. Cost utilization has also not changed significantly for patients with NIDCM (*p* > 0.05). However, patients with ICM demonstrated an increasing median cost of hospitalization from $47,059 (IQR: $33,795) in 2016 to $51,121 (IQR: $35,962) in 2020 (*p* < 0.001).

**FIGURE 5:**
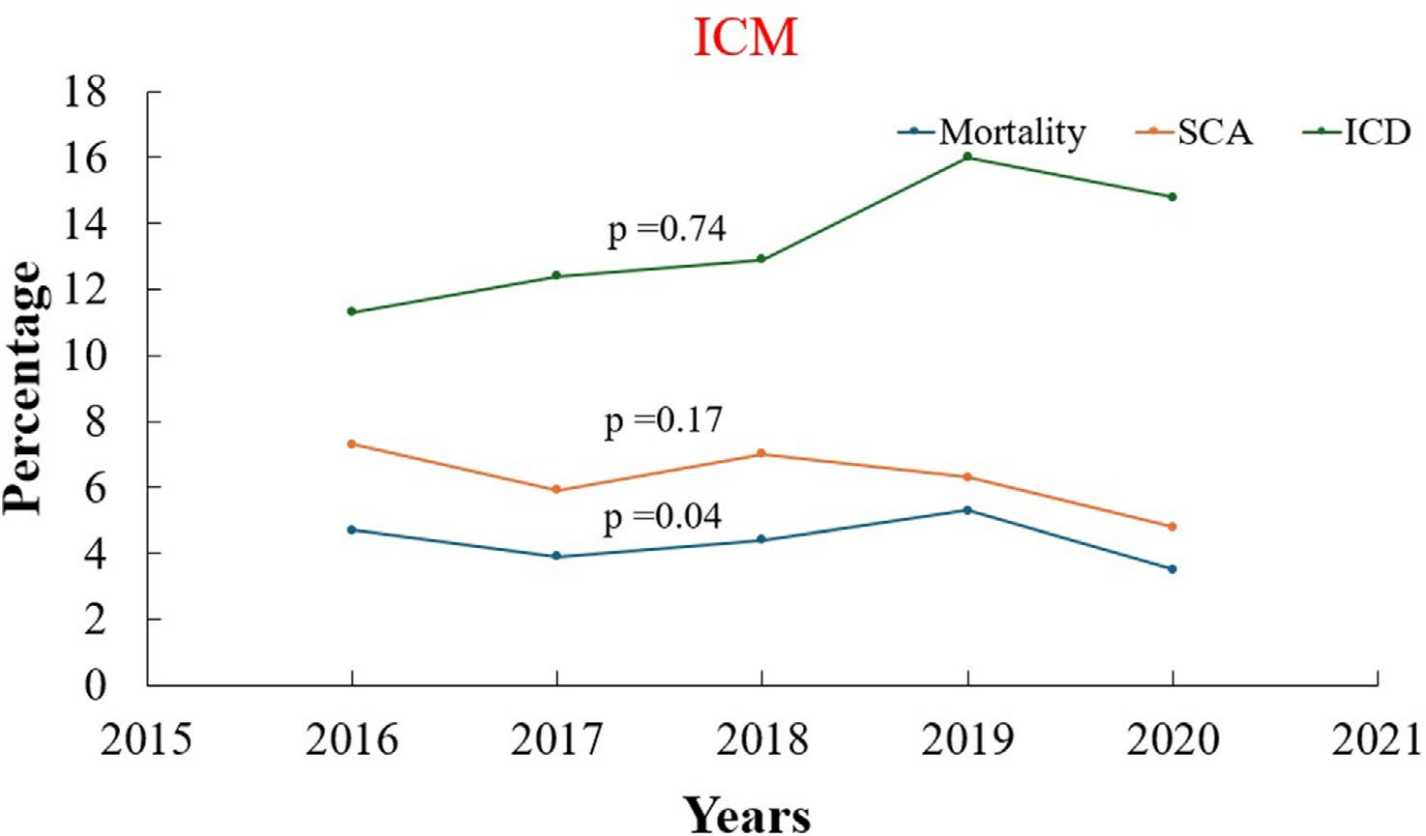
Outcome trends in ischemic cardiomyopathy. ICD, implantable cardioverter defibrillator; ICM, ischemic cardiomyopathy; SCA, sudden cardiac arrest.

**FIGURE 6:**
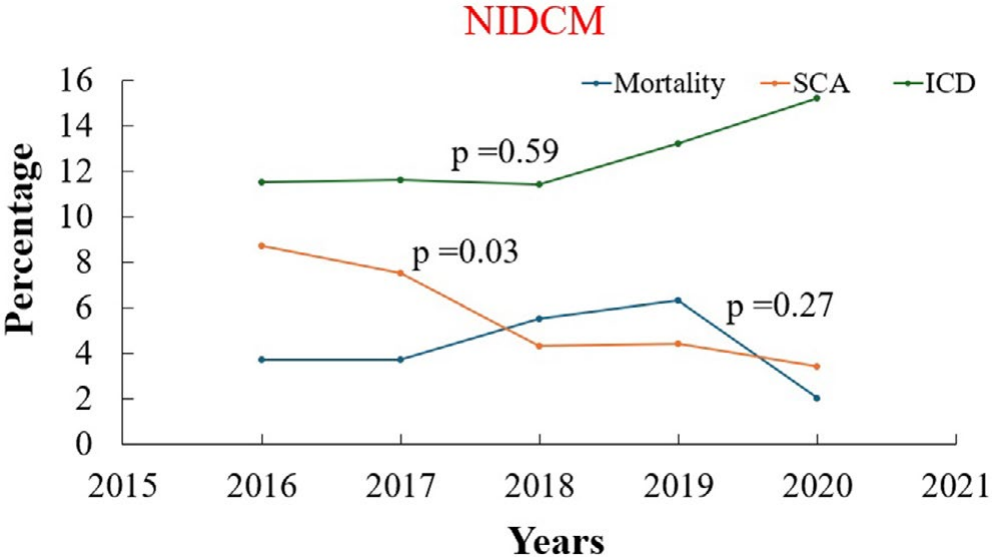
Outcome trends in non‐ischemic dilated cardiomyopathy. ICD, implantable cardioverter defibrillator; NIDCM, non‐ischemic dilated cardiomyopathy, SCA, sudden cardiac arrest.

From 2016 to 2020, in‐hospital mortality associated with hospitalizations involving VT ablation showed a decline in both ICM (4.7%–3.5%) and NIDCM (3.7%–2%) patients, although this was not statistically significant (*p*‐trend > 0.05). The incidence of sudden cardiac arrest significantly decreased in patients with underlying NIDCM (8.7%–3.4%, *p*‐trend: 0.028). However, it has not changed significantly for patients with ICM (*p* > 0.05) (Figures 5 and 6). Co‐implantation of ICD in patients admitted for VT ablation demonstrated an uptrend in patients with ICM (11.3%–14.8%, *p*‐trend: 0.045), while no significant trend is observed for patients with NIDCM (*p*‐trend > 0.05) as shown in Table 6.

Prior defibrillation (aOR: 1.59, 95% CI: 1.24–2.04, *p* < 0.001) and prior pacemakers (aOR: 2.04, 95% CI: 1.09–3.82, *p* = 0.026) were significant predictors of 30‐day readmission (Table S6). Heart failure (aOR: 1.50, 95% CI: 1.11–2.02, *p* < 0.001) was a significant predictor of 90‐day readmission (Table S7).

## 4 Discussion

In this NRD (2016–2020)‐based retrospective propensity‐matched analysis of patients undergoing VT ablation with ICM versus NIDCM, we observed the following key findings: (1) Higher risk of SCA, MACE, cardiogenic shock and periprocedural bleeding in the ICM cohort but no difference in overall mortality rates. (2) Higher risk of pericardial complications in the NIDCM cohort. (3) Up‐trending costs of VT ablation in the ICM cohort over the study period. (4) Higher readmission rates in the NIDCM cohort. (5) Down‐trending mortality and SCA rates in both ICM and NIDCM cohorts over the study period.

In a single‐center retrospective study of 248 patients undergoing VT ablation, Gomes et al. did not observe any significant difference in long‐term mortality (median follow‐up 2.3 years) between ICM and NIDCM patients (22.5% vs. 16.9%, *p* = 0.245) (Anon 2025a). However, they noted a significantly lower VT‐free survival in NIDCM patients (53.5% vs. 69.0%, *p* = 0.037) (Anon 2025a). In a prospective Heart Centre of Leipzig VT (HELP‐VT) Study of 227 patients undergoing VT ablation, Dinov et al. noted similar rates of complications (vascular access related, complete AV block, arteriovenous thromboembolism, bleeding, tamponade, left ventricular [LV] lead malfunction, heart failure [CHF] worsening) and in‐hospital deaths between NIDCM and ICM patients (Anon 2025b). In a previous analysis of NRD (2016–2018), no significant differences between ICM and NIDCM groups were observed with regard to overall complications (7.0% vs. 7.3%, *p* = 0.701) or in‐hospital mortality (3.7% vs. 3.5%, *p* = 0.608). However, there was a higher rate of cardiac perforation/tamponade in NIDCM than ICM group (2.4% vs. 1.5%, *p* = 0.008) (Sciria et al. 2022). Similarly, our study noted a higher rate of pericardial complications in NIDCM patients. This can be attributed to more frequent epicardial ablations performed in NIDCM than in ICM patients, increasing the risk of damaging the pericardium (Shirai et al. 2019; Kanagaratnam et al. 2022). It is unclear why there are higher rates of SCA, MACE, and cardiogenic shock in the ICM cohort than in the NIDCM patients in our study. Rottner et al. conducted a long‐term follow‐up on 334 patients undergoing VT ablation. They noted a higher 10‐year mortality in ICM patients (62.4%). It was speculated that older age and higher co‐morbidity burden in the ICM group vs. NIDCM group could be the underlying etiology (Gomes et al. 2024). However, the data regarding the differences in outcomes still remains contradictory between different studies and remains scarce. Our study results further this quest to determine the risk factors of these differences between the two cohorts.

Between 2016 and 2018, the overall complication rates (8.7% in 2016 to 6.2% in 2018, *p*‐trend < 0.001) and index mortality (4.4% in 2016 to 3.4% in 2018, *p*‐trend = 0.004) had a significant decline but this difference was only statistically significant in the ICM group (complications: 8.8% in 2016 vs. 6.0% in 2018, *p*‐trend < 0.001; index mortality: 4.5% in 2016 vs. 3.4% in 2018, *p*‐trend = 0.009). There was a non‐statistically significant decline in the NIDCM group (complications: 8.1% in 2016 vs. 6.9% in 2018, *p*‐trend = 0.186; index mortality: 4.3% in 2016 vs. 3.4% in 2018, *p*‐trend = 0.225) (Sciria et al. 2022). Our study was able to achieve statistical significance in both the ICM and NIDCM groups. It showed a statistically significant decline in mortality and SCA, likely due to a larger cohort of included patients and over a more extended period, increasing the study's power. In the study by Sciria et al., there was a significantly higher 30‐day readmission rate among the ICM than the NIDCM group (19.9% vs. 17.6%, *p* = 0.025), with female sex (OR, 1.24 [95% CI, 1.07–1.42]), renal disease (OR, 1.23 [95% CI, 1.08–1.41]), and diabetes mellitus (OR, 1.17 [95% CI, 1.04–1.32]) being the independent predictors of readmission. ICM itself was not an independent predictor of readmission (Sciria et al. 2022). The lack of detailed information about the adjusted variables in the prior study limits our ability to assess the accuracy of their results. This is remarkably different in our study. We observed a higher readmission rate in the NIDCM cohort, with only prior defibrillation being an independent predictor of 30‐day readmission. This aligns with the higher complexity of VT ablation in NIDCM patients, leading to lower rates of complete success (no inducible VT) and VT‐free survival (Shirai et al. 2019; Dinov et al. 2014).

Our study also highlights the significant rise in costs of VT ablation, especially in the ICM cohort. The data regarding cost‐effectiveness is lacking. The Ventricular Tachycardia Ablation versus Escalated Antiarrhythmic Drug Therapy in Ischemic Heart Disease (VANISH) trial was the first trial‐based cost‐effectiveness analysis comparing VT ablation with antiarrhythmic drugs in ICM VT. The results suggested an overall higher cost ($65,126 vs. $60,269; difference: $4857; 95% CI: −$19,757 to $27,106) and greater quality‐adjusted life‐years (QALYs) (1.63 vs. 1.49; difference: 0.14; 95% CI: −0.20 to 0.46) with ablation compared to drug therapy. However, there was a significant cost‐effectiveness ($67,614 vs. $68,383; difference: −$769; 95% CI: −$35,330 to $27,092) in amiodarone‐refractory VT with greater QALY (1.48 vs. 1.26; difference: 0.22; 95% CI: −0.19 to 0.59) (Rottner et al. 2025). The results of our study pave the way for future studies to investigate whether these rising costs are translated to improved QALYs.

## 5 Strengths

The novelty of our study lies in being the most extensive database analysis of VT ablation outcomes. The propensity‐matched outcomes with a rigorous amount of adjusted co‐morbidities provide power to the study. The results of our research align with the existing literature for the most part and also provide new data that need investigation with future prospective studies.

## 6 Limitations

Like any other study, the analysis has limitations. They should be considered when interpreting the results. Given the administrative nature of the data, there can be errors in coding and documentation. Due to a lack of laboratory and echocardiographic parameters, we cannot determine the severity of the illness, which may have affected our study outcomes. Hence, the lack of information on the specific etiology of the NIDCM VT limits the interpretation of our study findings. The lack of patient‐level data such as clinical, pharmacological, procedural characteristics and quality of life metrics further limits our study outcomes. Selection bias is inherent in observational studies. The observational nature of the data allows us only to determine associations, and causality cannot be established. Unmeasured or confounding variables may have affected the results. The data representing in‐hospital outcomes and out‐of‐hospital cardiac events in emergency room and ambulatory settings are not available, which may further limit our study results.

## 7 Conclusion

VT ablation in NIDCM patients is associated with a higher risk of pericardial complications and higher readmission rates. On the other hand, VT ablation in ICM patients demonstrates a higher risk of SCA, MACE, and cardiogenic shock, along with up‐trending costs of hospitalizations. The mortality and SCA are down‐trending in both ICM and NIDCM VT ablations, likely a result of the advancements in cardiac electrophysiology.

## Author Contributions

**Sanchit Duhan:** writing – original draft, review and editing, visualization, software. **Shafaqat Ali:** formal analysis, data curation, writing – review and editing. **Manoj Kumar** and **Thannon Alsaeed:** writing – original draft. **Bijeta Keisham:** writing – original draft. **Sanjay S. Mehta, Naveed A. Adoni, Anuj Garg, Mbu Mongwa** and **Benjamin J. Rhee:** supervision, validation, writing – review and editing.

## Disclosure

The authors have nothing to report.

## Conflicts of Interest

The authors declare no conflicts of interest.

## Supporting information


**Table S1:** ICD‐10 codes for cohort identification and co‐morbidities.
**Table S2:**. Definition of major outcomes.
**Table S3:** Variables used for propensity score matching.
**Table S4:** Variables used for multivariate regression.
**Table S5:** Readmission rates due to heart failure on propensity matched cohort.
**Table S6:** Multivariate regression analysis for predictors of 30‐day readmission.
**Table S7:** Multivariate regression analysis for predictors of 90‐day readmission.
**Figure S1:** Median length of stay.
**Figure S2:** Flow chart of the study.
**Figure S3:** Study variables.

## Data Availability

The nationwide readmission database is publicly available (https://hcup‐us.ahrq.gov/nrdoverview.jsp).
