# Supplementary material for: Periprocedural Outcomes of VT Ablation in Ischemic Compared to Non‐Ischemic Dilated Cardiomyopathy
Source: Ann Noninvasive Electrocardiol. 2025 Nov 4;30(6):e70126. doi: 10.1111/anec.70126 (PMC12586346; doi:10.1111/anec.70126)
Supplement: Supplementary file 1 — Table S1: ICD‐10 codes for cohort identification and co‐morbidities. Table S2:. Definition of major outcomes. Table S3: Variables used for propensity score matching. Table S4: Variables used for multivariate regression. Table S5: Readmission rates due to heart failure on propensity matched cohort. Table S6: Multivariate regression analysis for predictors of 30‐day readmission. Table S7: Multivariate regression analysis for predictors of 90‐day readmission. Figure S1: Median length of stay. Figure S2: Flow chart of the study. Figure S3: Study variables. [file ANEC-30-e70126-s001.docx]

**Supplementary Appendix**

| Description | Content |  |
| --- | --- | --- |
| ICD-10 codes for cohort identification and co-morbidities | Table S1 |  |
| Definitions of outcomes of interest | Table S2 |  |
| Variables used for Propensity Score Matching | Table S3 |  |
| Variables used in multivariate logistics regression | Table S4 |  |
| Readmission Rates due to Heart Failure on Propensity Matched Cohort | Table S5 |  |
| Multivariate Regression Analysis for Predictors of 30-Day Readmission | Table S6 |  |
| Multivariate Regression Analysis for Predictors of 90-Day Readmission | Table S7 |  |
| Median Length of Stay | Figure S1 |  |
| Flow chart of the study. | Figure S2 |  |
| Study variables. | Figure S3 |  |

**Table S1**. ICD-10 codes for cohort identification and co-morbidities

| **Study variables** | **ICD-10 Code** |
| --- | --- |
| VT | I47.2, I47.02 |
| ICM | I25.5 |
| Dilated Cardiomyopathy | I42.0 |
| History of Prior CABG | Z951 |
| History of prior PCI | Z955 |
| Obstructive CAD | I2510, I2511, I256, I2581, I2582 |
| Stroke | I63.XX |
| MCS | 5A02110, 5A02210, 5A0211D, 02HA3RZ, 5A02116, 5A0221D, 5A1522F 5A1522G, 5A1522H, 5A15A2F, 5A15A2G, 5A15A2H, |
| AKI | N170, N171, N172, N178, N179, N1 |
| Pericardiac Complications | Cardiac Tamponade: I314, Hemopericardium: I312, Pericarditis: I30, I31, Pericardial Drainage: 0W9D30Z, 0W9D3ZX, 0W9D3ZZ |
| Myocardial Infarction | I2101, I2102, I2109, I2111, I2119, I2121, I2129, I213, I214 |
| Sudden Cardiac Arrest | I46, I97, 029, 004, 007 |
| ICD | 02H43KZ, 02H63KZ, 02H64KZ, 02H70KZ, 02H73KZ, 02H74KZ, 02HK3KZ, 02HK4KZ, 02HL0KZ, 02HL3KZ, 02HL4KZ, 0JH608Z, 0JH609Z, 0JH60FZ, 0JH638Z, 0JH639Z, 0JH63FZ, 0JH808Z, 0JH809Z, 0JH838Z, 0JH839Z |
| VT Ablation | 02583ZZ, 02573ZZ, 02553ZZ, 02563ZZ |
| Cardiogenic shock | R570, T8111XA, T8111XS |
| Acute CHF | I5021, I5023, I5031, I5033, I5041, I5043 |
| LVAD Utilization | 02HA0QZ, 02HA3QZ, 02HA4QZ, 02PA0RZ, 02WA0QZ |
| ECMO Utilization | 5A1522F, 5A1522G, 5A1522H, 5A1522J, 5A15223 |
| Cardiac Transplantation | 02YA0Z0, 02YA0Z1, 02YA0Z2 |
| Intubation | 0BH17EZ, 0BH18EZ |
| Obesity | E66, Z683, Z684, R939, Z6854, 09921 |
| Hypertension | I10, I1150, I1151, I1152, I1158, I1159 |
| Diabetes Mellitus | E08-E13 family |
| Alcohol use | F101, F1010, F1011, F1012, F10120, F10121, F10129, F1014, F1015, F10150, F10151, F10159, F1018, F10180, F10181, F10182, F10188, F1019, F102, F1020, F1021, F1022, F10220, F10221, F10229, F1023, F10230, F10231, F10232, F10239, F1024, F1025, F10250, F10251, F10259, F1026, F1027, F1028, F10280, F10281, F10282, F10288, F1029, F10920, F10921, F10929, F1094, F1095, F10950, F10951, F10959, F1096, F1097, F1098, F10980, F10981, F10982, F10988, F1099 |
| Atrial fibrillation/flutter | I48 family |
| Heart failure | I50, I501, I502, I5020, I5021, I5022, I5023, I503, I5030, I5031, I5032, I5033, I504, I5040, I5041, I5042, I5043, I508, I5081, I50810, I50811, I50812, I50813, I50814, I5082, I5083, I5084, I5089, I509 |
| Chronic obstructive pulmonary disease | J449 |
| Smoker | F17, Z87.891 |
| Chronic kidney disease stage ≥ 3 | N183, N184, N185, E082, E132, I12, I13 |
| ESRD | N186, Z992, Z4931, Z4901 |
| Anemia | D50, D51, D52, D53, D55, D56, D57, D58, D59, D60, D61, D62, D63, D64, D46.0, D46.1, D46.2, D46.4, O99.0 |
| Obstructive sleep apnea | G47.33 |
| History of stroke | I69.3, Z86.73 |
| History of prior ICD | Z95.810 |
| Peripheral vascular disease | E08.5, E09.5, E10.5, E11.5, E13.5, I73, T82.856, Z98.62, Z95.820, I25.2, I25.83 |

**All codes are available at “**[**https://www.icd10data.com/**](https://www.icd10data.com/)**”** AMI Acute Myocardial Infarction, pLVAD: Percutaneous Left Ventricular Assist Device, VA-ECMO: Veno-arterial Extracorporeal Membrane Oxygenation, IABP: Intra-aortic Baloon Pump, CABG: Coronary Artery Bypass Grafting, PCI: Percutaneous Coronary Intervention, PCA: Percutaneous Coronary Angiogram MCS: Mechanical Circulatory Support, ESRD: End Stage Renal Disease, BMI: Body Mass Index, CHF: Congestive heart failure, ICD: Implantable cardioverter defibrillator

**Table S2.** Definition of Major Outcomes

| Outcomes | Definition |
| --- | --- |
| Mortality | All causes of death, including cardiovascular causes such as sudden cardiac death, death due to acute myocardial infarction, heart failure or cardiogenic shock, and non-cardiovascular causes. |
| VT | Patients admitted with ventricular tachycardia upon arrival or patients who subsequently developed ventricular tachycardia during the index hospitalization |
| ICM | Ischemic cardiomyopathy (ICM) is a condition in which heart muscle dysfunction or heart failure occurs due to coronary artery disease (CAD) and reduced blood supply to the heart. |
| NIDCM | Non-ischemic dilated cardiomyopathy (NIDCM) refers to heart muscle disease that leads to ventricular dysfunction and heart failure without significant coronary artery disease (CAD) or myocardial ischemia as the primary cause. |
| MACE | Major Adverse Cardiovascular and Cerebrovascular Event; MACCE includes in-hospital mortality, myocardial infarction, cardiogenic shock and stroke. |
| Acute CHF | Heart Failure in the setting of ventricular tachycardia |
| Cardiogenic Shock | Shock resulting from primary failure of the heart in its pumping function, such as in myocardial infarction, severe ischemic cardiomyopathy, mechanical obstruction, or compression of the heart in the setting of VT |
| LOS | The entire length of stay the patient spent in the hospital during the admission. |
| Cost of hospitalization | The total adjusted amount that the hospital got reimbursed for providing their services for the duration of hospitalization |

LOS: Length of Stay, VT: Ventricular tachycardia, ICM: ischemic cardiomyopathy; NIDCM: Non-ischemic dilated cardiomyopathy

**Table S3.** Variables used for Propensity Score Matching

| Variables included in Propensity Matching |
| --- |
| Age |
| Gender |
| Rehab Transfer |
| Resident |
| Elective/Non-elective admissions |
| Hospital Bed Size |
| Hospital Location & Teaching Status |
| Hospital Region |
| Weekend Admission |
| Payer |
| Diabetes |
| Obesity |
| Hypertension |
| Hyperlipidemia |
| Smoker |
| CKD Stage >3 |
| ESRD |
| Prior PCI |
| Prior Defibrillator |
| OSA |
| Pulmonary Disease |
| Pulmonary HTN |
| Hypothyroidism |
| Anemia |
| Liver disease |
| COVID-19 |

Abbreviations: OSA; Obstructive sleep apnea, CKD; Chronic kidney disease, ESRD; End-Stage renal disease, PCI: Percutaneous coronary intervention, HTN: Hypertension

**Table S4.** Variables used for Multivariate Regression

| Variables included in Propensity Matching |
| --- |
| Age |
| Gender |
| Rehab Transfer |
| Resident |
| Elective/Non-elective admissions |
| Hospital Bed Size |
| Hospital Location & Teaching Status |
| Hospital Region |
| Weekend Admission |
| Payer |
| Diabetes |
| Obesity |
| Hypertension |
| Hyperlipidemia |
| Smoker |
| CKD Stage >3 |
| ESRD |
| Prior PCI |
| Prior Defibrillator |
| OSA |
| Pulmonary Disease |
| Pulmonary HTN |
| Hypothyroidism |
| Anemia |
| Liver disease |
| COVID-19 |

Abbreviations: OSA; Obstructive sleep apnea, CKD; Chronic kidney disease, ESRD; End-Stage renal disease, PCI: Percutaneous coronary intervention, HTN: Hypertension

| **Table S5:** Readmission Rates due to Heart Failure on Propensity Matched Cohort | | | |
| --- | --- | --- | --- |
| **Readmission Rates on Propensity Matched Cohort** | | | |
| **30 day Readmissions** | **VT in ICM** | **VT in NIDCM** | **P-value** |
|  | **N = 1093** | **N = 1093** |  |
|  | **N (%)** | **N (%)** |  |
| **Readmits** | 170 (15.6) | 105 (9.6) | <0.001 |
| **90 day Readmissions** | **VT in ICM** | **VT in NIDCM** | **P-value** |
|  | **N = 885** | **N = 885** |  |
|  | **N (%)** | **N (%)** |  |
| **Readmits** | 281 (31.8) | 222 (25.1) | 0.002 |
| **180 day Readmissions** | **VT in ICM** | **VT in NIDCM** | **P-value** |
|  | **N = 1591** | **N = 1591** |  |
|  | **N (%)** | **N (%)** |  |
| **Readmits** | 608 (38.2) | 700 (44) | 0.001 |
| **Abbreviations:** VT: Ventricular Tachycardia; ICM: Ischemic Cardiomyopathy; NIDCM: Non-ischemic dilated cardiomyopathy | | | |

| **Table S6**: Multivariate Regression Analysis for Predictors of 30-Day Readmission | | | |
| --- | --- | --- | --- |
| **Predictors of Readmission - 30d** | **aOR** | **95 % CI** | **P-value** |
| **Comorbidites** | | | |
| VT in NIDCM | 0.98 | 0.70-1.37 | 0.908 |
| Female Gender | 1.15 | 0.84-1.59 | 0.384 |
| Diabetes | 1.24 | 0.98-1.55 | 0.068 |
| Hyperlipidemia | 1.23 | 0.96-1.57 | 0.106 |
| Hypertension | 0.99 | 0.72-1.37 | 0.969 |
| Smoker | 0.9 | 0.71-1.15 | 0.411 |
| CKD Stage over 3 | 1.03 | 0.78-1.36 | 0.839 |
| ESRD | 1.41 | 0.72-2.74 | 0.311 |
| Prior PPM | 2.04 | 1.09-3.82 | 0.026 |
| Prior Defibrillation | 1.59 | 1.24-2.04 | <0.001 |
| OSA | 0.99 | 0.74-1.33 | 0.967 |
| Pulmonary Disease | 1.25 | 0.95-1.66 | 0.114 |
| Pulmonary Hypertension | 1.12 | 0.64-1.97 | 0.693 |
| Hypothyroidism | 1.09 | 0.79-1.51 | 0.596 |
| Anemia | 0.91 | 0.48-1.72 | 0.772 |
| Pneumonia | 0.58 | 0.26-1.31 | 0.189 |
| Liver Disease | 0.47 | 0.12-1.88 | 0.285 |
| Heart Failure | 1.09 | 0.77-1.54 | 0.618 |
| **Admission Day of The Week** | | | |
| Weekend Admit | 1.2 | 0.91-1.60 | 0.198 |
| **Insurance Type *** | | | |
| Medicaid | 0.84 | 0.48-1.48 | 0.54 |
| Private Insurance | 0.73 | 0.54-0.97 | 0.032 |
| Self-Pay | 0.45 | 0.12-1.67 | 0.232 |
| No Charge | 3.95 | 0.77-20.25 | 0.1 |
| Other | 0.49 | 0.23-1.02 | 0.056 |
| **Hospital Characteristics: Hospital Bed Size †** | | | |
| Medium | 0.6 | 0.35-1.03 | 0.063 |
| Large | 0.6 | 0.37-0.96 | 0.034 |
| **Hospital Characteristics: Hospital urban-rural designation ‡** | | | |
| Small Metropolitan | 1.08 | 0.84-1.39 | 0.562 |
| Micropolitan Area | 0.93 | 0.42-2.06 | 0.857 |
| **Hospital Characteristics: Control/Ownership of Hospital §** | | | |
| Private, not-profit | 1.06 | 0.73-1.52 | 0.767 |
| Private, invest-own | 1.06 | 0.64-1.77 | 0.814 |
| * Compared to Medicare | | | |
| † Compared to the hospitals with small bed size | | | |
| ‡ Compared to large metropolitan areas with a least 1 million residents | | | |
| § Compared to Government/Non-Federal Hospitals | | | |
| **Abbreviations:** VT in NIDCM: Ventricular Tachycardia in Non-ischemic dilated cardiomyopathy; CKD: Chronic Kidney Disease; ESRD: End Stage Renal Disease; PPM: Permanent Pacemaker Placement; OSA: Obstructive Sleep Apnea; aOR: Adjusted Odds Ratio; CI: Confidence Interval | | | |

| **Table S7**: Multivariate Regression Analysis for Predictors of 90-Day Readmission | | | |
| --- | --- | --- | --- |
| **Predictors of Readmission - 90d** | **aOR** | **95 % CI** | **P-value** |
| **Comorbidites** | | | |
| VT in NIDCM | 1 | 0.74-1.34 | 0.994 |
| Female Gender | 0.91 | 0.66-1.24 | 0.543 |
| Diabetes | 1.18 | 0.96-1.45 | 0.107 |
| Hyperlipidemia | 1.3 | 1.02-1.65 | 0.03 |
| Hypertension | 0.83 | 0.64-1.08 | 0.175 |
| Smoker | 0.87 | 0.71-1.07 | 0.19 |
| CKD Stage over 3 | 1.16 | 0.90-1.50 | 0.257 |
| ESRD | 1.34 | 0.75-2.40 | 0.324 |
| Prior PPM | 1.51 | 0.82-2.80 | 0.186 |
| Prior Defibrillation | 1.22 | 0.98-1.51 | 0.073 |
| OSA | 1.15 | 0.84-1.56 | 0.385 |
| Pulmonary Disease | 1.31 | 1.01-1.69 | 0.041 |
| Pulmonary Hypertension | 1.05 | 0.67-1.65 | 0.815 |
| Hypothyroidism | 1.11 | 0.82-1.50 | 0.501 |
| Anemia | 1.29 | 0.82-2.00 | 0.268 |
| Pneumonia | 1.04 | 0.59-1.82 | 0.897 |
| Liver Disease | 0.31 | 0.09-1.05 | 0.061 |
| Heart Failure | 1.5 | 1.11-2.02 | 0.008 |
| **Admission Day of The Week** | | | |
| Weekend Admit | 1.07 | 0.83-1.38 | 0.615 |
| **Insurance Type *** | | | |
| Medicaid | 1.08 | 0.68-1.70 | 0.74 |
| Private Insurance | 0.66 | 0.49-0.89 | 0.006 |
| Self-Pay | 0.6 | 0.25-1.45 | 0.254 |
| No Charge | 2.48 | 0.30-20.22 | 0.396 |
| Other | 0.62 | 0.30-1.28 | 0.2 |
| **Hospital Characteristics: Hospital Bed Size †** | | | |
| Medium | 0.6 | 0.37-0.97 | 0.038 |
| Large | 0.69 | 0.44-1.08 | 0.108 |
| **Hospital Characteristics: Hospital urban-rural designation ‡** | | | |
| Small Metropolitan | 1.01 | 0.81-1.26 | 0.938 |
| Micropolitan Area | 1.04 | 0.50-2.13 | 0.924 |
| **Hospital Characteristics: Control/Ownership of Hospital §** | | | |
| Private, not-profit | 1.04 | 0.78-1.39 | 0.806 |
| Private, invest-own | 0.98 | 0.62-1.53 | 0.914 |
| * Compared to Medicare | | | |
| † Compared to the hospitals with small bed size | | | |
| ‡ Compared to large metropolitan areas with a least 1 million residents | | | |
| § Compared to Government/Non-Federal Hospitals | | | |
| **Abbreviations:** VT in NIDCM: Ventricular Tachycardia in Non-ischemic dilated cardiomyopathy; CKD: Chronic Kidney Disease; ESRD: End Stage Renal Disease; PPM: Permanent Pacemaker Placement; OSA: Obstructive Sleep Apnea; aOR: Adjusted Odds Ratio; CI: Confidence Interval | | | |

**Figure S1.** Median Length of Stay


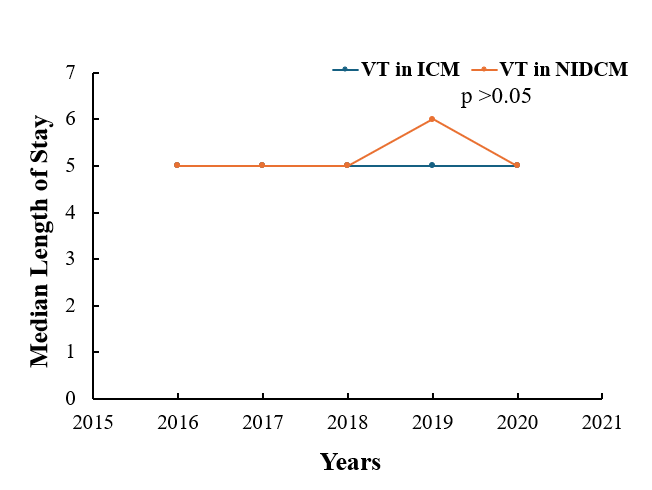
Abbreviations: VT-ventricular tachycardia, ICM-ischemic cardiomyopathy, NIDCM-non-ischemic dilated cardiomyopathy

**Figure S2.** Flow chart of the study.


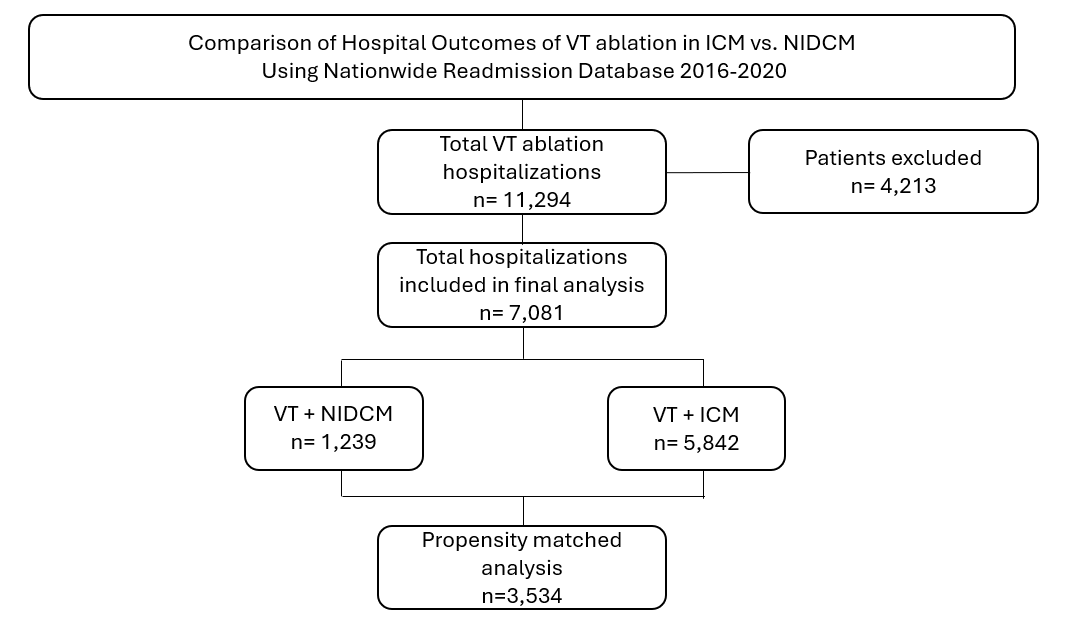


**Abbreviations**: VT-ventricular tachycardia, NIDCM-nonischemic dilated cardiomyopathy, ICM-ischemic cardiomyopathy

**Figure S3.** Study variables.


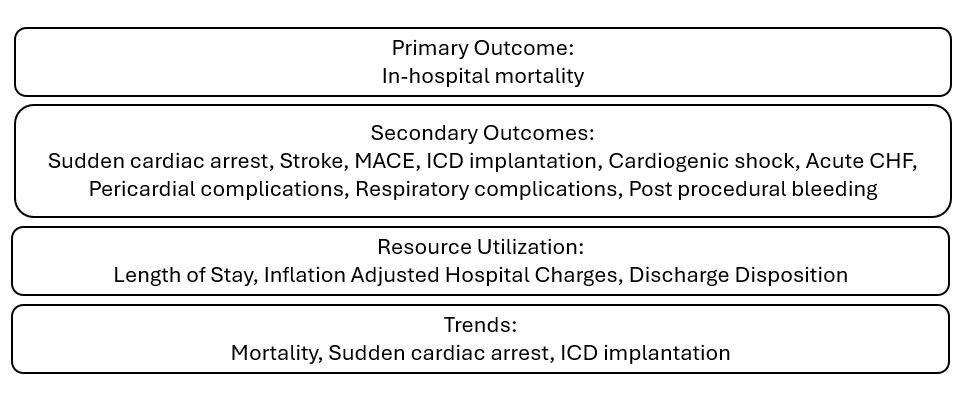


**Abbreviations:** ICD-implantable cardioverter defibrillator, SCA-sudden cardiac arrest, MACE-major adverse cardiovascular events, CHF-congestive heart failure
